# Impact of denosumab on bone high-resolution peripheral quantitative computed tomography in hemodialysis patients: a retrospective longitudinal observational study

**DOI:** 10.1093/ckj/sfag182

**Published:** 2026-09-21

**Authors:** Benjamin Batteux, Ana Pimentel, Agnes Ostertag, Tilman B Drüeke, Martine Cohen-Solal, Pablo Antonio Ureña Torres

**Affiliations:** Department of Pharmacology, Amiens University Medical Center, Amiens, France; MP3CV Laboratory, EA7517, Jules Verne University of Picardie, Amiens, France; AURA Paris, Saint Ouen, France; INSERM U1132, Université Paris-Cité and Department of Rheumatology, Lariboisière Hospital, Paris, France; CESP, Inserm U-1018, Université Paris-Sud, Villejuif, France; Department of Biostatistics, Versailles Saint-Quentin-en-Yvelines University (Paris-Ile-de-France-Ouest University, UVSQ), Villejuif, France; INSERM U1132, Université Paris-Cité and Department of Rheumatology, Lariboisière Hospital, Paris, France; AURA Paris, Saint Ouen, France

**Keywords:** chronic kidney disease–mineral bone disorder, denosumab, dialysis, high-resolution peripheral quantitative computed tomography

## Abstract

**Background and hypothesis:**

Denosumab increases bone mineral density (BMD) in dialysis patients, but the effects on bone microarchitecture using high-resolution peripheral quantitative computed tomography (HRpQCT) and the response factors to treatment are unknown.

**Methods:**

We analyzed, at a single center between 2014 and 2024, 19 consecutive patients on hemodialysis having a longitudinal HRpQCT monitoring at the distal tibia and radius. After excluding the presence of osteomalacia based on bone biopsies and ensuring well-controlled parathyroid hormone levels, nine patients with recent fractures were treated with denosumab. Changes in HRpQCT parameters observed over time were analyzed within the denosumab group and were compared to a group of a nontreated patients, using linear mixed models for repeated measures. Baseline histomorphometric parameters were analyzed as predictors of changes in HRpQCT parameters over time in denosumab-treated patients.

**Results:**

During a median [maximum–minimum] follow-up period of 2.1 [1.0–3.5] years, the nine denosumab-treated patients had a significant improvement of (i) bone volume-to-total volume ratio at the distal radius (+1.63% [95% confidence interval: 1.11–3.31] per year, *P* = .037) and tibia (+1.13% [0.10–2.28], *P* = .049), (ii) trabecular volumetric BMD at the distal radius (+1.58 [1.07–3.30], *P* = .039) and tibia (+0.95% [0.11–1.97], *P* = .039), (iii) trabecular number at the distal radius (+1.86% [0.45–3.28], *P* = .017), and (iv) trabecular separation at the distal radius (−1.83% [−3.16 to −0.50], *P* = .014). These changes were also significant when compared with those of the untreated group. Changes in cortical parameters did not change significantly over time. At most, changes in trabecular volumetric BMD at the radius were negatively associated with osteoid volume.

**Conclusion:**

Denosumab improves HRpQCT trabecular bone parameters without significant effect on cortical parameters in dialysis patients.

## Graphical Abstract

**Graphical Abstract:**
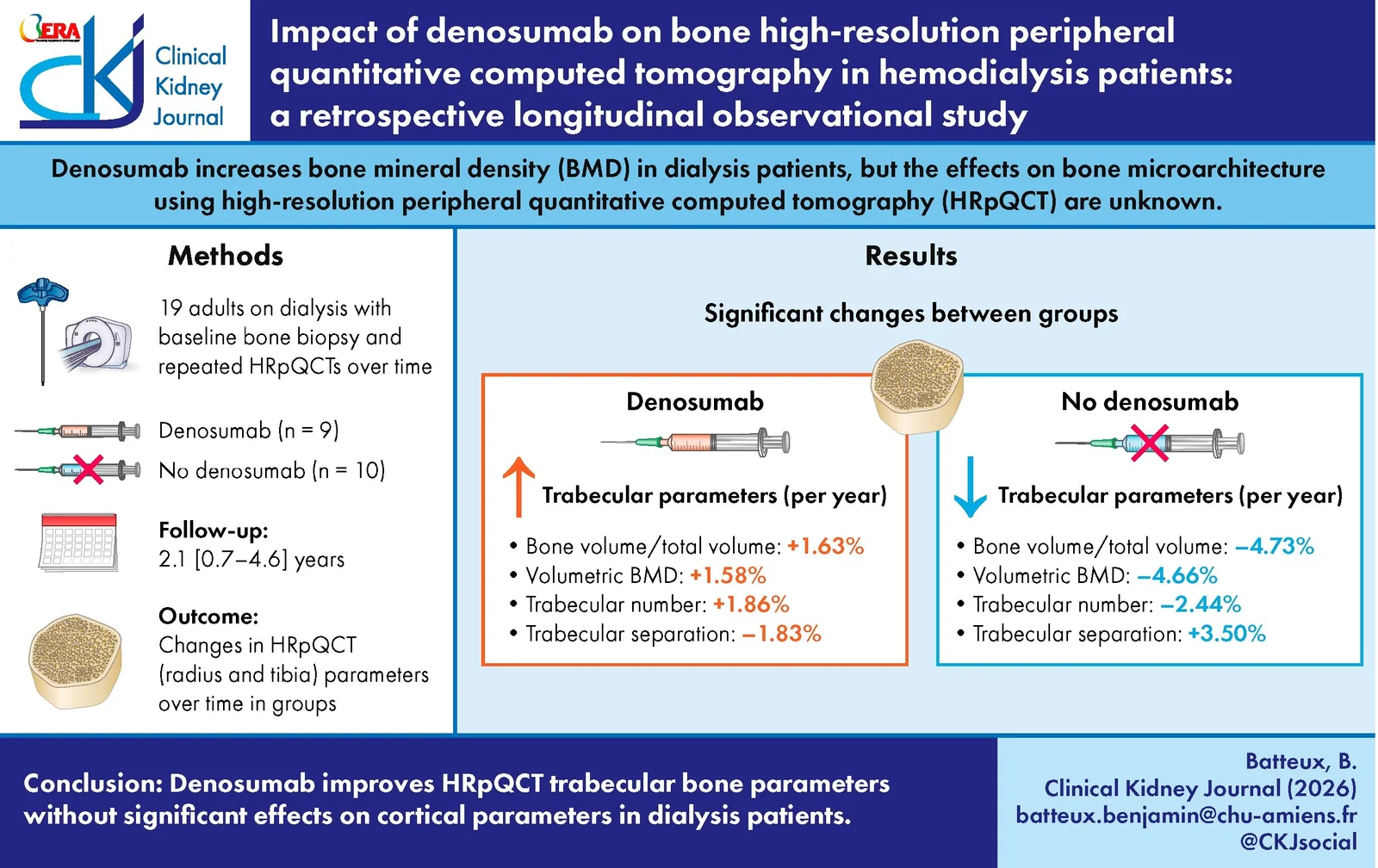

KEY LEARNING POINTS **What was known:**

- Dual-energy X-ray absorptiometry (DXA) is recommended for fracture risk assessment in chronic kidney disease stages 3a–5D patients.
- Unlike DXA, high-resolution peripheral quantitative computed tomography (HRpQCT) is not subject to certain artifacts and enables the assessment of bone microarchitecture.
- The effects of denosumab on bone microarchitecture using HRpQCT have not yet been reported in patients on hemodialysis.

**This study adds:**

- Denosumab improved HRpQCT trabecular bone microarchitecture in hemodialysis patients with recent fractures and well-controlled parathyroid hormone levels.
- No changes in HRpQCT cortical parameters were observed over time in denosumab-treated patients.
- Optimal response to denosumab seems to occur when bone mineralization defects are absent.

**Potential impact:**

- HRpQCT should be used and integrated into routine practice to provide systematic assessment and monitoring of dialysis patients treated with denosumab.

## INTRODUCTION

Chronic kidney disease (CKD) stages 3–5D are linked to complications like CKD–mineral and bone disorder, which includes CKD-associated osteoporosis and CKD-associated cardiovascular disease [1]. Renal osteodystrophy (ROD), now considered part of CKD-associated osteoporosis, is marked by abnormal bone turnover and mineralization due to disturbances in calcium, phosphate, parathyroid hormone (PTH), and vitamin D. The updated Kidney Disease Improving Global Outcomes (KDIGO) guideline recommends the use of bone mineral density (BMD) assessed by dual-energy X-ray absorptiometry (DXA) for fracture risk assessment in CKD 3a–5D patients, though interpretation is challenging due to factors like vascular calcification and variable bone quality [1]. High-resolution peripheral quantitative computed tomography (HRpQCT), may offer a more accurate assessment in this population, especially for patients on dialysis, while DXA’s correlation with bone histomorphometry remains weak [2–6].

ROD significantly increases the risk of fragility fractures, as demonstrated by the Dialysis Outcomes and Practice Patterns Study [7]. The Chronic Renal Insufficiency Cohort (CRIC) study reported a combined incidence of hip and vertebral fractures of 2.4 per 1000 person-years [95% confidence interval (CI), 2.0–2.9] among patients with CKD over an 11-year follow-up period [8]. A recent consensus statement on the management of vertebral fractures in patients with CKD stages G4–G5D highlights that vertebral fractures represent a common manifestation of skeletal fragility in this population, affecting up to 50% of individuals [9]. Management primarily aims to normalize serum levels of calcium, phosphate, vitamin D, and PTH. Among antiresorptive therapies, denosumab may be considered, particularly since bisphosphonates are contraindicated in advanced CKD, once serum calcium and PTH are within recommended target ranges [1, 10]. Denosumab has been shown to increase BMD in dialysis patients, including those with severe secondary hyperparathyroidism (SHPT) [11–13]; however, these studies generally featured short follow-up periods [11–19]. Although, indications for antifracture therapies based on bone turnover rate require further clarification, current evidence suggests that controlling SHPT prior to denosumab initiation in CKD patients is essential [20]. As such, denosumab should be reserved for patients with fractures and PTH levels within recommended KDIGO targets [10].

To better understand the impact of denosumab on bone health in hemodialysis patients with PTH levels within target ranges who have suffered fragility fractures, we set out to evaluate changes in bone microarchitecture using HRpQCT. Additionally, we explored whether specific histomorphometric parameters could serve as predictors of the microarchitectural changes induced by denosumab.

## MATERIALS AND METHODS

### Study design and participants

We performed a single-center retrospective longitudinal observational study of adult patients (aged 18 and over) with end-stage kidney disease on intermittent hemodialysis therapy who had osteoporosis evaluation by bone HRpQCT measurement at Lariboisière University Medical Center, Paris, France between 1 April 2014 and 31 September 2024. Of the 32 patients who had HRpQCT assessment during this period, 19 had longitudinal HRpQCT follow-up and were therefore included in this study. Among them, 9 patients received subcutaneous injections of denosumab 60 mg every 6 months, and 10 patients did not receive denosumab. Denosumab was administered according to our protocol, i.e. in patients with fragility fracture who had a serum PTH level in a range 2–9 higher than the upper limit of normal (ULN), and normal bone mineralization as demonstrated by bone histomorphometry [10]. Denosumab was not used otherwise. The end of follow-up period was either 31 September 2024, or (i) 1 year after kidney transplantation or parathyroidectomy, as these interventions lead to substantial changes in bone microarchitecture, or (ii) in patients treated with denosumab, 6 months after the last drug administration in case of treatment withdrawal. The study protocol included a HRpQCT at the right distal tibia and at the ultradistal and proximal radius of the arm without an arteriovenous-fistula at baseline and subsequently every year, a DXA examination at the hip and lumbar spine at baseline and annually during follow-up, and circulating biochemical markers of mineral and bone metabolism.

### Bone microarchitecture and BMD measurements

Volumetric BMD (vBMD) and microarchitectural parameters were assessed at the right distal tibia and on the nondominant, arteriovenous fistula-free arm at distal radius site by HRpQCT (XtremeCT, Scanco Medical AG, Brüttisellen, Switzerland), as previously described [21]. BMD was measured once yearly by DXA with an iDXA device (GE Medical Systems Lunar, Madison, WI) at lumbar spine (L1–L4), hip (total hip and femoral neck), and radius (ultradistal and proximal). Areal BMD (aBMD, g/cm²), Z-scores, and T-scores were evaluated with reference to the French population. We also used the World Health Organization definitions of aBMD categories: normal bone density (T-score ≥ −1), osteopenia (T-score −1 to −2.5), and osteoporosis (T-score < −2.5). Results are presented in accordance with the guidelines from the International Society for Clinical Densitometry.

### Clinical and biochemical markers of mineral and bone metabolism

The clinical variables recorded included age, sex, body–mass index (BMI), ethnic group, time on hemodialysis, diabetes mellitus, previous parathyroidectomy, fracture history, etiology of CKD, treatments received, and bone histomorphometric parameters. Blood samples were collected in the morning under fasting conditions between two hemodialysis sessions to determine total plasma calcium, phosphate, PTH, 25-OH vitamin D (25OHD), and bone biomarkers: total alkaline phosphatase (tALP), bone-specific alkaline phosphatase (Bs-ALP), C-terminal telopeptide of type 1 collagen (CTX), procollagen 1 intact N terminal propeptide (P1NP), and tartrate-resistant acid phosphate 5B (TRAP-5b).

### Bone histomorphometry

Participants underwent double doxycycline labeling for bone histomorphometry analysis. The protocol consisted of 3 days of doxycycline (100 mg twice daily), followed by a doxycycline-free interval of 10 days, and then doxycycline (500 mg twice daily) for an additional 3 days. The final 2D static and dynamic histomorphometry results of mild SHPT-related bone disease, osteitis fibrosa, adynamic bone disease, and osteomalacia, respectively, were categorized according to the “turnover/mineralization/volume (TMV)” classification [22, 23]. Bone histomorphometry parameters were determined using the HISTOLAB^®^ software (MICROVISION^®^, France). Mineral apposition rate (MAR, µm/day) was measured and bone formation rate (BFR/BS, µm^3^/µm^2^/day) was calculated [23]. A value of 0.1 mm/day was used for MAR when double labels were undetectable in the different sections [24].

### Outcomes

The primary outcome was changes in HRpQCT parameters over time at distal radius and tibia in denosumab-treated patients (denosumab group). The secondary outcomes were (i) changes in HRpQCT parameters over time at distal radius and tibia as a function of bone histomorphometric parameters [BFR/BS, osteoid surface (OS/BS), and osteoid volume (OV/BV)] in denosumab-treated patients, (ii) changes in HRpQCT parameters over time in the denosumab group versus patients not treated with denosumab (no-denosumab group).

### Statistical methods

In a descriptive analysis, categorical variables were expressed as the number (percentage), and continuous variables were expressed as the mean ± standard deviation (SD), the median [interquartile range (IQR)], or the median (range), depending on data distribution. The Shapiro–Wilk test was used to determine whether data were normally distributed. In bivariate analyses comparing groups (i.e. denosumab exposure or not), continuous variables were compared using Student’s *t*-test or a Wilcoxon’s rank sum test (depending on data distribution), and categorical variables were compared using a chi-square test or Fisher’s exact test.

In the study patients who had repeated HRpQCT measurements over time, linear mixed models for repeated measures (MMRMs) were built to assess the influence of denosumab exposure on changes in HRpQCT parameters over time. An MMRM was built for each of the two measurement sites (distal tibia and radius) and for each of the HRpQCT parameters studied: trabecular bone volume to tissue volume fraction (BV/TV; %), trabecular volumetric density (Dtrab; mg/cm^3^), trabecular number (TbN; 1/mm), trabecular thickness (TbTh; mm), trabecular separation (TbSp; mm), trabecular distribution (Tb.1/N.SD; tmn), cortical volumetric density (Dcomp; mg/cm^3^), cortical thickness (CtTh; mm), and cortical perimeter (CtPm; mm). Changes in HRpQCT parameters over time were modeled as percentage changes relative to baseline. Because of the limited number of subjects, models included a random intercept only, without a random slope for time, to avoid overfitting and potential model instability. In a first analysis, restricted to the denosumab group, the effect of time on the continuous outcome variable (i.e. the HRpQCT parameter of interest) was evaluated. Time was modeled as a continuous variable to assess a linear temporal trend. Consequently, these models were build using only time as fixed effect and a subject-specific random intercept.

In a second analysis, treated and untreated patients were compared to evaluate whether changes in outcomes (i.e. the HRpQCT parameters) over time differed between groups. The models included time, treatment group, and their interaction as fixed effects, and a subject-specific random intercept. Then, these models were adjusted to control for potential intergroup confounding factors. To avoid potential overfitting, inverse probability‐of‐treatment weighting (IPTW) was used. Furthermore, the use of IPTW ensures that the distribution of the probability of receiving one treatment or another is similar across the weighted population [25]. A propensity score (PS) was estimated using a logistic regression model. Treatment assignment (denosumab or not) was regressed against age at baseline, sex, BMI at baseline, time on hemodialysis, diabetes mellitus, PTH, and Bs-ALP levels at baseline. The history of fracture could not be included in the PS because all denosumab-treated patients had a fracture, which would have resulted in convergence issues. To avoid excluding patients from the analyses, histomorphometric parameters were not included, as bone biopsy data were not available for all patients. For this reason, PTH and Bs-ALP levels, reflecting bone remodeling [26], were included in the models. The goal of the PS is to create balance, not to achieve good fit [25]. The balance of the PS was assessed by examining the standardized mean differences. For regression adjustment to be trustworthy, the absolute standardized differences of means should be <0.25 [27], which was the case here. Hence, MMRMs including denosumab exposure, time and an interaction term of treatment by time as fixed-effects, and using IPTW were performed for each HRpQCT parameters. Patients in the denosumab group were weighted by the reciprocal of their PS, and patients in the control group were weighted by one minus the reciprocal of their PS.

The same methodology was used to analyze the influence of denosumab exposure on (i) DXA parameters (BMD, T-score, and Z-score) over time at lumbar spine, total hip, femoral neck, one-third radius, and ultradistal radius, and (ii) biochemical markers of mineral and bone metabolism (serum ionized calcium, phosphate, CTX, PTH, 25OHD, tALP, Bs-ALP, TRAP-5b, and P1NP).

MMRMs were also built to analyze bone histomorphometric parameters (BFR, OV/BV, and OS/BS) as predictive factors of changes in HRpQCT parameters over time in denosumab-treated patients. Fixed effects included the histomorphometric parameter of interest and time.

All MMRMs were estimated using restricted maximum likelihood. Degrees of freedom for fixed effects were approximated using the Satterthwaite method. Statistical significance was defined as a two-sided *P*-value < .05. All analyses were performed using R software (version 4.0.5, R Foundation for Statistical Computing, Vienna, Austria).

This study was conducted using data obtained from the usual standard of care of our hospital. After informed consent, agreements were collected (https://www.aphp.fr/RGPD). Study for research was approved (codecoh number DC-2020-3976).

## RESULTS

### Study population

In the denosumab group (*N* = 9), the median age was 65.4 years, seven were menopausal women (Table 1). All these patients had a history of fracture: seven had one or several spinal fractures, one had a pelvic fracture, one had an ankle fracture, and one had rib fractures in addition to a spinal fracture. At baseline, the median PTH levels was 295 pg/ml. A bone biopsy was performed in seven patients, with a median [minimum–maximum] interval of 7.5 [3.5–12.2] months before the first denosumab injection. Histomorphometric evidence of mild SHPT-related bone disease was found in four of them, and osteitis fibrosa in three. Two patients did not require a bone biopsy, as there was no suspicion of osteomalacia (Table 1 and Supplementary Table S1). None of them received denosumab or bisphosphonates prior to inclusion. Four of them received cinacalcet prior to denosumab, three received phosphate binders. All of them received vitamin D supplements during the follow-up period, and calcium supplements following denosumab injections.

**Table 1:** Baseline characteristics of the study population.

Baseline characteristics	Study population *N* = 19	No-denosumab group *N* = 10	Denosumab group *N* = 9	*P*-value
Demographic data				
Age (years)	70.3 [62.7–75.5]	70.3 [63.0–76.7]	65.4 [63.2–73.6]	.903
Sex (female)	13 (68.4)	6 (60.0)	7 (77.8)	.735
BMI (kg/m²)	24.8 [20.8–26.8]	25.6 [ 22.5–28.1]	22.7 [20.6–24.8]	.253
Ethnic group				1.000
Caucasian	17 (89.5)	9 (90.0)	8 (88.9)	
Black	2 (10.5)	1 (10.0)	1 (11.1)	
Medical history				
Diabetes mellitus	5 (26.3)	2 (20.0)	3 (33.3)	.891
Parathyroidectomy	6 (31.6)	3 (30.0)	3 (33.3)	1.000
Time on hemodialysis (years)	6.4 [2.4–11.4]	6.0 [2.7–13.5]	6.4 [2.2–11.3]	.780
Fracture	13 (68.4)	5 (50.0)	9 (100)	.185
Laboratory data at baseline				
Serum ionized calcium (RV: 2.12–2.52 mmol/l)	1.10 [1.04–1.18]	1.10 [1.03–1.24]	1.10 [1.08–1.20]	.917
Serum phosphate (RV: 0.81–1.58 mmol/l)	1.26 [1.03–1.45]	1.21 [0.91–1.52]	1.34 [1.23–1.45]	.810
PTH (RV: 10–65 pg/ml)	375.5 [172.2–659.2]	470.0 [197.0–700.0]	295.0 [164.0–609.0]	.605
25OH D (RV: 50–136 ng/l)	42.9 [28.4–52.2]	32.6 [30.4–43.2]	48 [26.9–62.5]	.460
Bs-ALP (RV: 4–25 µg/l)	47.1 [19.4–70.8]	64.7 [30.5–71.0]	30.5 [18.8–60.0]	.573
TRAP-5b (RV < 4 IU/l)	3.55 [2.63–5.98]	4.90 [3.33–5.98]	3.45 [2.63–4.70]	.683
TMV classification (bone histomorphometry)	*N* = 12	*N* = 5	*N* = 7	.264
Mild hyperparathyroidism	5 (41.7)	1 (20.0)	4 (57.1)	
Osteitis fibrosa	4 (33.3)	1 (20.0)	3 (42.9)	
Adynamic bone disease	1 (8.3)	1 (20.0)	0	
Osteomalacia	2 (16.7)	2 (40.0)	0	

Continuous variables are quoted as the median [interquartile range], categorical variables are quoted as the number (percentage). med, median; RV, reference values.

In the no-denosumab group (*N* = 10), the median age was 70.3 years, six were menopausal women (Table 1). All six had a history of fracture: three had one or several spinal fractures, one had a pelvic fracture, one had a pelvic and a spinal fracture, and one had a pelvic and a shoulder fracture. At baseline, the median PTH levels was 470 pg/ml. A bone biopsy was performed in these five patients: one had mild SHPT-related bone disease, one had osteitis fibrosa, one had adynamic bone disease, and two had osteomalacia (Table 1 and Supplementary Table S1). During the study period, eight patients were treated with cinacalcet, including those with very-high PTH levels, four with phosphate binders, six with calcium supplements, and all with vitamin D supplements.

The clinical, biochemical, and histomorphometric characteristics at baseline did not differ significantly between the groups, although the denosumab group had more fractures and did not present the same type of ROD compared to the no-denosumab group (Table 1 and Supplementary Table S1).

HRpQCT and DXA parameters did not differ significantly between groups at baseline (Supplementary Tables S2 and S3).

### Changes in HRpQCT parameters over time

The median [minimum–maximum] length of HRpQCT follow-up was 2.1 [1.0–3.5] years in the denosumab group, and 2.1 [0.7–4.6] years in the untreated group.

In the denosumab group, at the distal radius, BV/TV increased significantly by +1.63% per year, Dtrab by +1.58%, and TbN by +1.86%, whereas TbSp decreased significantly by −1.83% per year (Fig. 1 and Table 2). At the distal tibia, BV/TV increased significantly by +1.13% [0.10–2.28] (*P* = .049), and Dtrab by +0.95% [0.11–1.97] per year (*P* = .039, Table 2, and Supplementary Figure S1). Most of these findings were observed when compared with the no-denosumab group (Fig. 1, Supplementary Figures S1 and S2, and Table 2). No significant changes were found in cortical parameters over time at the distal radius or tibia (Table 2 and Supplementary Figure S2).

**Figure 1:**
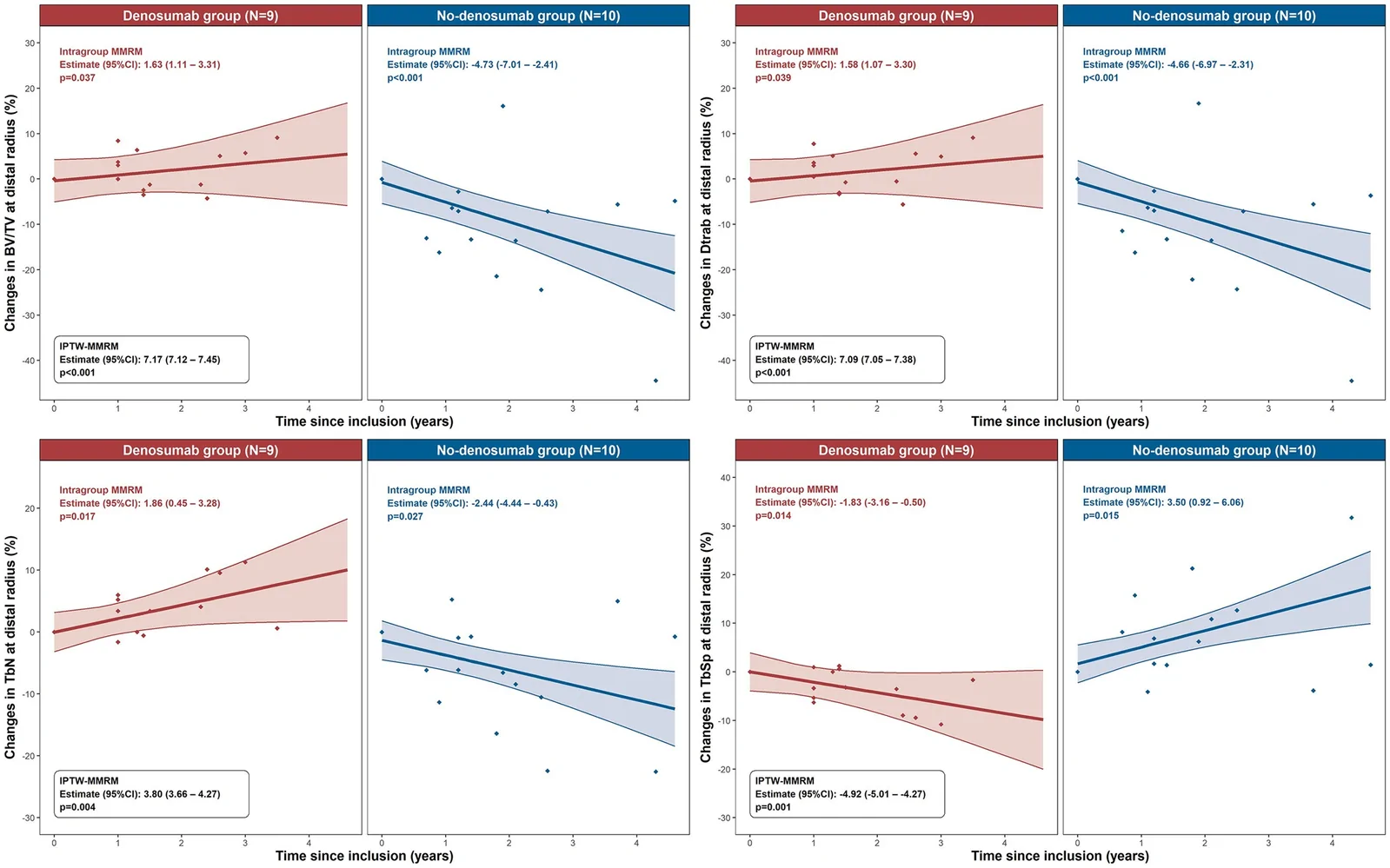
Changes in HRpQCT trabecular parameters at distal radius in groups (MMRM plots). *N*, number of patients with repeated HRpQCT measurements over time.

**Table 2:** Changes in HRpQCT parameters over time in groups (MMRMs).

	Intragroup analyses				Denosumab group versus No-denosumab group			
HRpQCT	Denosumab group*N* = 9		No-denosumab group*N* = 10		Nonadjusted MMRMs		IPTW-MMRMs	
parameters	Estimate (95% CI)*	*P*-value	Estimate (95% CI)*	*P*-value	Estimate (95% CI)**	*P*-value	Estimate (95% CI)**	*P*-value
Distal radius								
BV/TV	1.63 (1.11–3.31)	.037	−4.73 (−7.01 to −2.41)	<.001	6.33 (3.32–9.32)	<.001	7.17 (7.12–7.45)	<.001
Dtrab	1.58 (1.07–3.30)	.039	−4.66 (−6.97 to −2.31)	<.001	6.20 (3.16–9.23)	<.001	7.09 (7.05–7.38)	<.001
Dcomp	−0.64 (−1.67 to −0.31)	.207	−2.69 (−3.72 to −1.69)	<.001	2.02 (0.57–3.44)	0.010	2.03 (2.00–2.16)	.006
CtTh	−0.63 (−3.19–1.67)	.599	−6.46 (−9.71 to −3.39)	.001	5.54 (1.39–9.63)	0.014	5.31 (5.24–5.76)	.010
CtPm	0.31 (−0.04–0.71)	.101	−0.02 (−0.35–0.64)	.882	0.37 (−0.06–0.84)	0.109	0.42 (0.31–0.52)	.071
TbN	1.86 (0.45–3.28)	.017	−2.44 (−4.44 to −0.43)	.027	4.30 (1.69–6.91)	0.003	3.80 (3.66–4.27)	.004
TbTh	0.09 (−2.66–2.83)	.952	−2.63 (−4.76 to −0.57)	.023	2.85 (−0.54–6.29)	0.114	4.43 (4.29–4.97)	.014
TbSp	−1.83 (−3.16 to −0.50)	.014	3.50 (0.92–6.06)	.015	−5.33 (−8.5 to −2.15)	0.003	−4.92 (−5.01 to −4.27)	.001
Tb1/N.SD	−2.18 (−6.51–1.90)	.311	7.32 (2.70–11.93)	.006	−10.01 (−16.62–3.41)	0.006	−8.20 (−8.67 to −7.75)	.012
Distal tibia								
BV/TV	1.13 (0.10–2.28)	.049	−4.36 (−6.85–1.77)	.003	5.50 (2.31–8.55)	0.002	5.58 (5.31–5.72)	<.001
Dtrab	0.95 (0.11–1.97)	.039	−4.40 (−6.83 to −1.87)	.003	5.33 (2.24–8.29)	0.002	5.46 (5.24–5.62)	<.001
Dcomp	0.07 (−0.16–0.32)	.563	−2.07 (−4.11 to −0.07)	.060	2.15 (−0.17–4.58)	0.088	1.66 (−0.97–2.35)	.210
CtTh	1.42 (−1.10–3.73)	.251	−5.84 (−13.91–1.88)	.157	7.13 (−2.01–16.77)	0.148	5.32 (−1.13–10.77)	.295
CtPm	−0.13 (−0.035–0.08)	.236	−0.34 (−0.56 to −0.13)	.005	0.21 (−0.11–0.54)	0.216	0.19 (−0.81–0.30)	.240
TbN	0.26 (−3.42–3.56)	.882	−1.12 (−7.92–4.39)	.512	1.06 (−4.09–5.88)	0.677	0.52 (−2.16–2.69)	.811
TbTh	−0.44 (−10.18–9.67)	.931	−0.82 (−5.17–3.43)	.711	0.37 (−9.36–10.52)	0.942	5.51 (4.85–7.54)	.296
TbSp	−8.80 (−20.16–2.55)	.142	14.25 (0.28–28.22)	.059	−23.12 (−42.37 to −3.86)	0.027	−26.88 (−28.37 to −23.92)	.004
Tb.1/N.SD	−744 (−25.17–9.63)	.405	41.86 (8.45–75.27)	.023	−49.84 (−91.79 to −7.89)	0.029	−56.07 (−27.10 to −48.61)	.004

*The reported estimates correspond to the time-fixed effect.

** The reported estimates correspond to the fixed effect of time * treatment interaction.

Regarding the influence of baseline bone histomorphometric parameters on changes in HRpQCT parameters over time, OV/BV was negatively correlated with changes in Dtrab at the distal radius (estimate 95% CI: −0.92 [−1.65 to −1.17], *P* = .033, Table 3). The other histomorphometric parameters were not significantly associated with changes in the other HRpQCT parameters over time, at the distal radius or tibia (Tables 3 and 4).

**Table 3:** Changes in HRpQCT parameters over time at radius according to BFR/BS, OS/BS, and OV/BV in denosumab-treated patients (MMRMs).

	BFR/BS*N* = 7		OS/BS*N* = 7		OV/BV*N* = 7	
	Estimate (95% CI)	*P*-value	Estimate (95% CI)	*P*-value	Estimate (95% CI)	*P*-value
BV/TV	−0.20 (−0.55–0.15)	.297	−0.16 (−0.45–0.13)	.323	−0.82 (−1.57 to −0.07)	.055
Dtrab	−0.17 (−0.53–0.19)	.495	−0.20 (−0.50–0.09)	.211	−0.92 (−1.65 to −1.17)	.033
Dcomp	0.24 (0.07–0.42)	.018	0.03 (−0.19–0.25)	.780	0.15 (−0.46–0.74)	.650
CtTh	0.28 (−0.47–1.02)	.517	0.13 (−0.46–0.70)	.687	0.33 ( −1.29–1.88)	.711
CtPm	−0.02 (−0.15–0.10)	.729	0.00 (−0.10–0.09)	.960	−0.02 (−0.27–0.25)	.911
TbN	−0.02 (−0.32–0.28)	.925	0.03 (−0.20–0.26)	.804	0.06 (−0.59–0.73)	.861
TbTh	−0.17 (−0.81–0.47)	.672	−0.21 (−0.68–0.27)	.482	−1.00 (−2.28–0.26)	.153
TbSp	0.04 (−0.23–0.30)	.833	0.00 (−0.22–0.22)	.971	0.02 (−0.59–0.64)	.960
Tb.1/N.SD	−0.89 (−2.13–0.38)	.272	−0.12 (−1.29–1.04)	.855	−0.85 (−3.98–2.25)	.624

**Table 4:** Changes in HRpQCT parameters over time at tibia according to BFR/BS, OS/BS, and OV/BV in denosumab-treated patients (MMRMs).

	BFR/BS*N* = 7		OS/BS*N* = 7		OV/BV*N* = 7	
HRpQCT parameters	Estimate (95% CI)	*P*-value	Estimate (95% CI)	*P*-value	Estimate (95% CI)	*P*-value
BV/TV	0.17 (0.00–0.34)	.082	−0.04 (−0.17–0.09)	.618	−0.15 (−0.53–0.23)	.502
Dtrab	0.13 (−0.04–0.31)	.165	−0.02 (−0.16–0.11)	.760	−0.13 (−0.51–0.24)	.545
Dcomp	−0.08 (−0.14 to −0.03)	.012	0.02 (−0.05–0.08)	.650	0.05 (−0.11–0.22)	.561
CtTh	−0.16 (−0.6–0.04)	.278	0.03 (−0.20–0.26)	.800	0.19 (−0.43–0.81)	.595
CtPm	0.031 (−0.02–0.08)	.373	0.02 (−0.01–0.06)	.286	0.06 (−0.04–0.16)	.341
TbN	1.26 (0.50–2.01)	.008	0.10 (−0.81–1.02)	.852	0.43 (−2.04–2.95)	.754
TbTh	−2.28 (−4.54 to −0.01)	.082	0.01 (−1.91–1.98)	.994	1.33 (−3.95–6.86)	.659
TbSp	−1.46 (−4.25–1.33)	.383	0.99 (−0.77–2.70)	.331	1.12 (−4.20–6.31)	.701
Tb.1/N.SD	−3.05 (−6.73–0.58)	.192	1.35 (−1.36–3.97)	.384	3.24 (−4.37–10.68)	.500

### Changes in DXA and biochemical parameters over time

In the denosumab group, BMD increased significantly at the lumbar spine, total hip, and femoral neck. These improvements were also observed when compared with the no-denosumab group (Fig. 2 and Supplementary Table S4).

**Figure 2:**
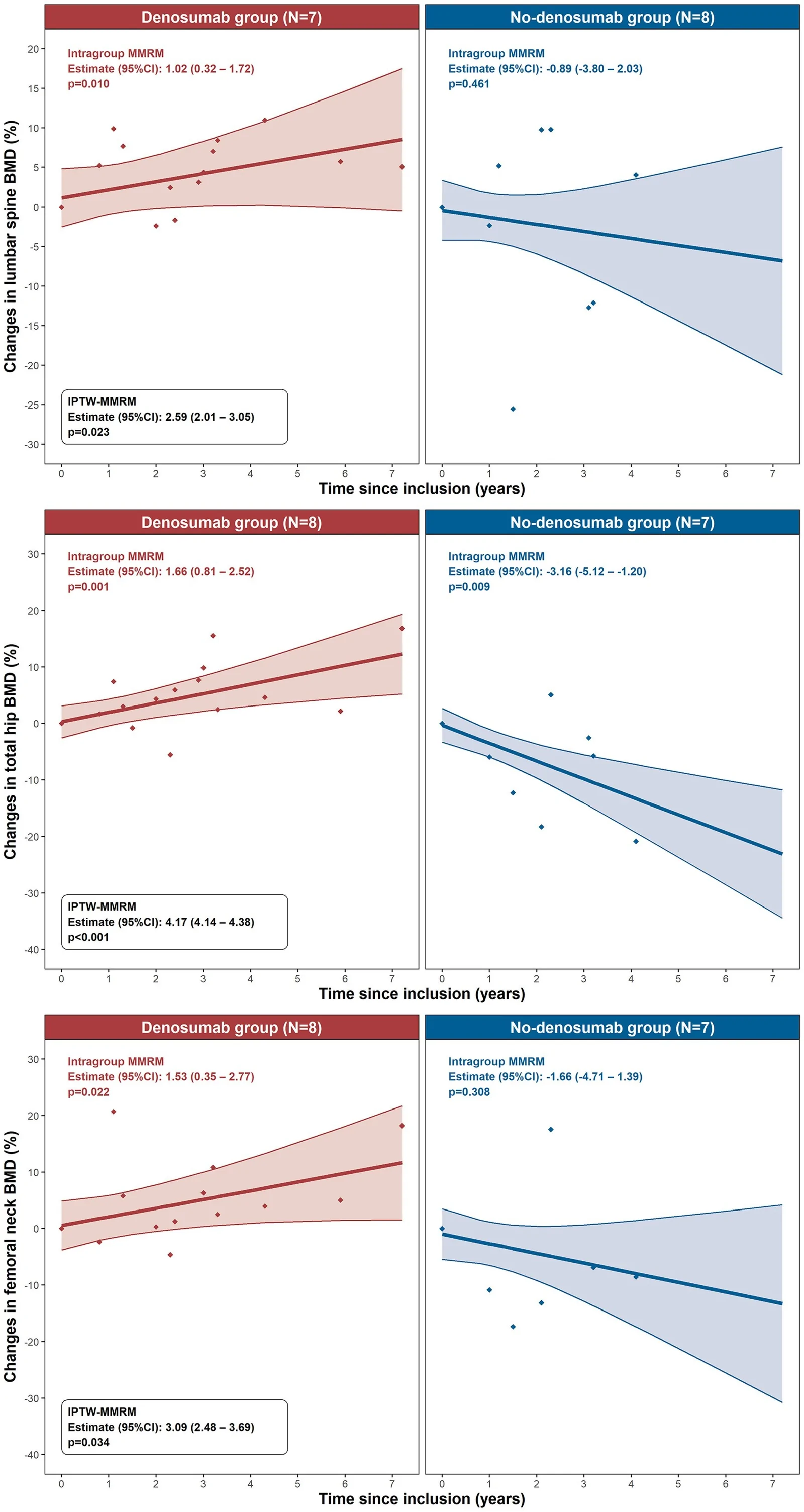
Changes in BMD (DXA) in groups (MMRM plots). *N*, number of patients with repeated HRpQCT measurements over time.

Regarding biochemical parameters, denosumab exposure was associated with a significant decrease in PTH, Bs-ALP, and P1NP levels over time (Fig. 3 and Supplementary Table S5).

**Figure 3:**
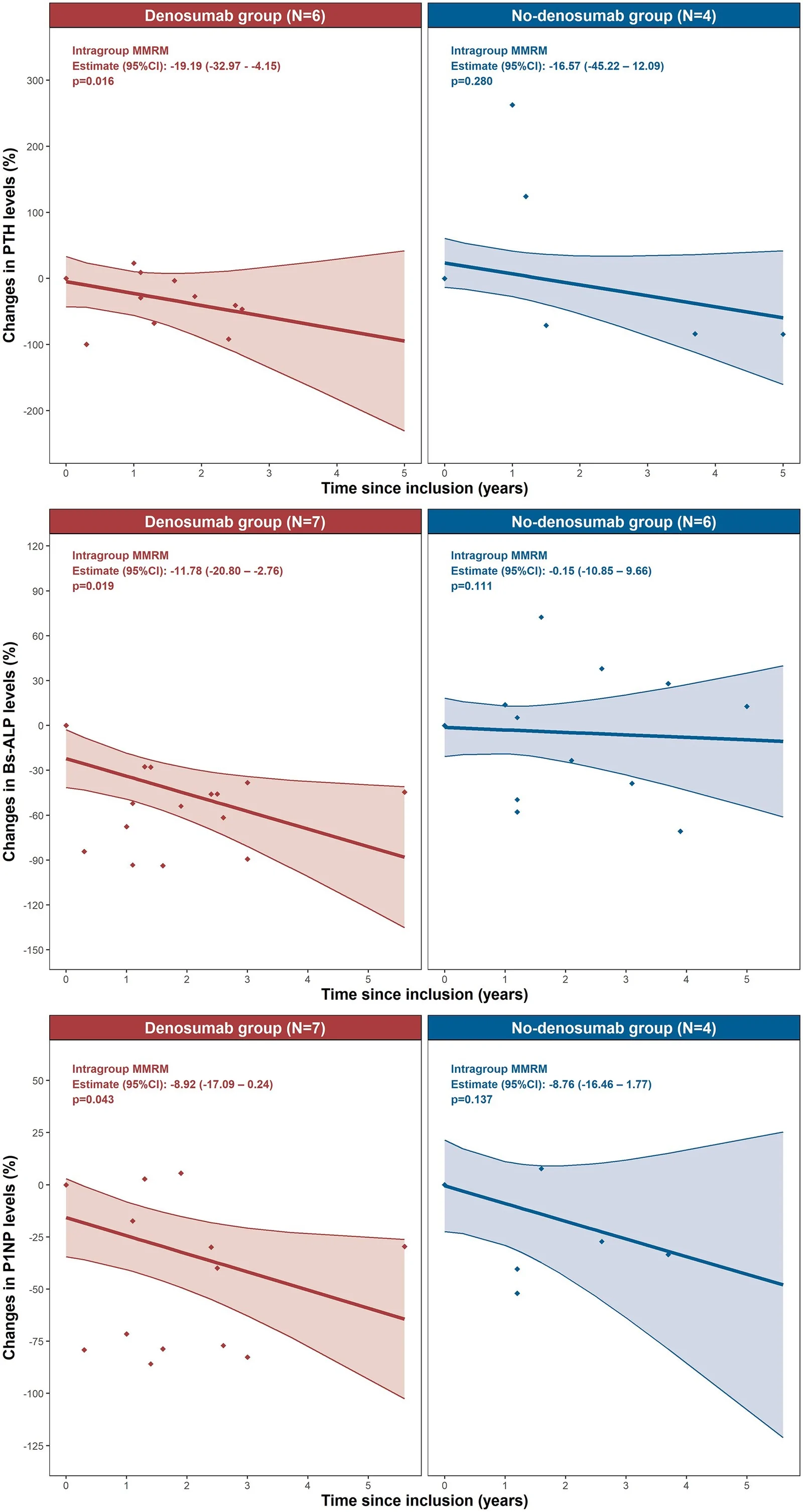
Changes in biochemical parameters in groups (MMRM plots). *N*, number of patients with repeated HRpQCT measurements over time.

## DISCUSSION

To our knowledge, this study is the first to longitudinally assess both trabecular and cortical bone changes using HRpQCT in dialysis patients treated with denosumab, with baseline histomorphometric date guiding interpretation. The principal finding demonstrates that denosumab significantly enhanced trabecular vBMD and bone volume/total volume at both tibia and radius, as well as increased TbN at the radius, in hemodialysis presenting with fractures, well-controlled PTH levels, and mild SHPT-related bone disease or osteitis fibrosa. Moreover, changes in trabecular vBMD at the radius were inversely associated with baseline OV/BV, as determined by bone histomorphometry. Compared to a group of hemodialysis patients not receiving denosumab and exhibiting a spectrum of bone phenotype, those treated with denosumab showed greater improvements in trabecular microarchitectural. t

The 2017 KDIGO guideline [28] and its current version [1] recommends BMD testing to assess fracture risk if results are likely to impact treatment decision. This recommendation was based on four longitudinal studies including a very small number of dialysis patients, and DXA was performed at the femoral neck in just two studies [29–32]. Furthermore, DXA evaluation at lumbar spine should be interpreted with caution in such patients, as results may be overestimated due frequent deformities, degenerative changes, and vascular calcifications [10]. Changes in DXA parameters at lumbar spine over time may also be uninterpretable due to overestimations from progressing vascular calcification, whether or not these patients are treated with denosumab [16, 33, 34].

Most studies investigating the effects of denosumab on bone health in dialysis patients have utilized DXA to assess BMD [11–19]. Notably, one study using three-dimensional DXA to evaluate trabecular and cortical vBMD at the hip in denosumab-treated dialysis patients found increases in both compartments [35]. In our study, we observed significant improvements in BMD not only at the total hip but also at the lumbar spine in the denosumab group, both when assessed longitudinally and in comparison with untreated controls, thereby reinforcing the finding of prior research [14–19]. However, we did not detect significant BMD changes at the radius, which may be attributable to the limited sample size or potentially to site-specific differences in bone response to therapy.

HRpQCT is a suitable tool for evaluating effects of denosumab on bone, as it allows precise analysis of cortical and trabecular vBMD, microarchitecture, thickness, and porosity. Supporting this, a study by Marques *et al*. showed agreement between HRpQCT and bone biopsy parameters for trabecular and cortical bone, and a significant inverse correlation between cortical vBMD at radius and various circulating bone biomarkers in dialysis patients [36]. Our study demonstrates improvements in trabecular microarchitectural parameters (BV/TV, Dtrab, TbN, and TbSp) in denosumab-treated dialysis patients, in contrast to continuous bone loss in untreated patients.

This study is the first to longitudinally evaluate microarchitectural bone changes in dialysis patients treated with denosumab, using baseline histomorphometry data for detailed interpretation. Notably, we observed a significant inverse association between baseline OV/BV and Dtrab at the radius, suggesting that underlying mineralization defects may attenuate the skeletal response to denosumab, as its mechanism of increasing BMD fundamentally depends on the bone’s capacity to undergo secondary mineralization. In the FREEDOM trial, denosumab leads to an increase of mineralization by completion of secondary mineralization mainly in the osteonal and interstitial bone structural units [37]. In conditions where mineralization is severely impaired, such as osteomalacia, denosumab is unable to convert unmineralized osteoid into mineralized bone. Consequently, we would recommend performing a tetracycline double-labeled bone biopsy prior to therapy initiation to identify such defects (Fig. 4). Importantly, in the present study, this negative association was not observed in other trabecular parameters, and these findings should be interpreted with caution in light of the study’s design and sample size.

**Figure 4:**
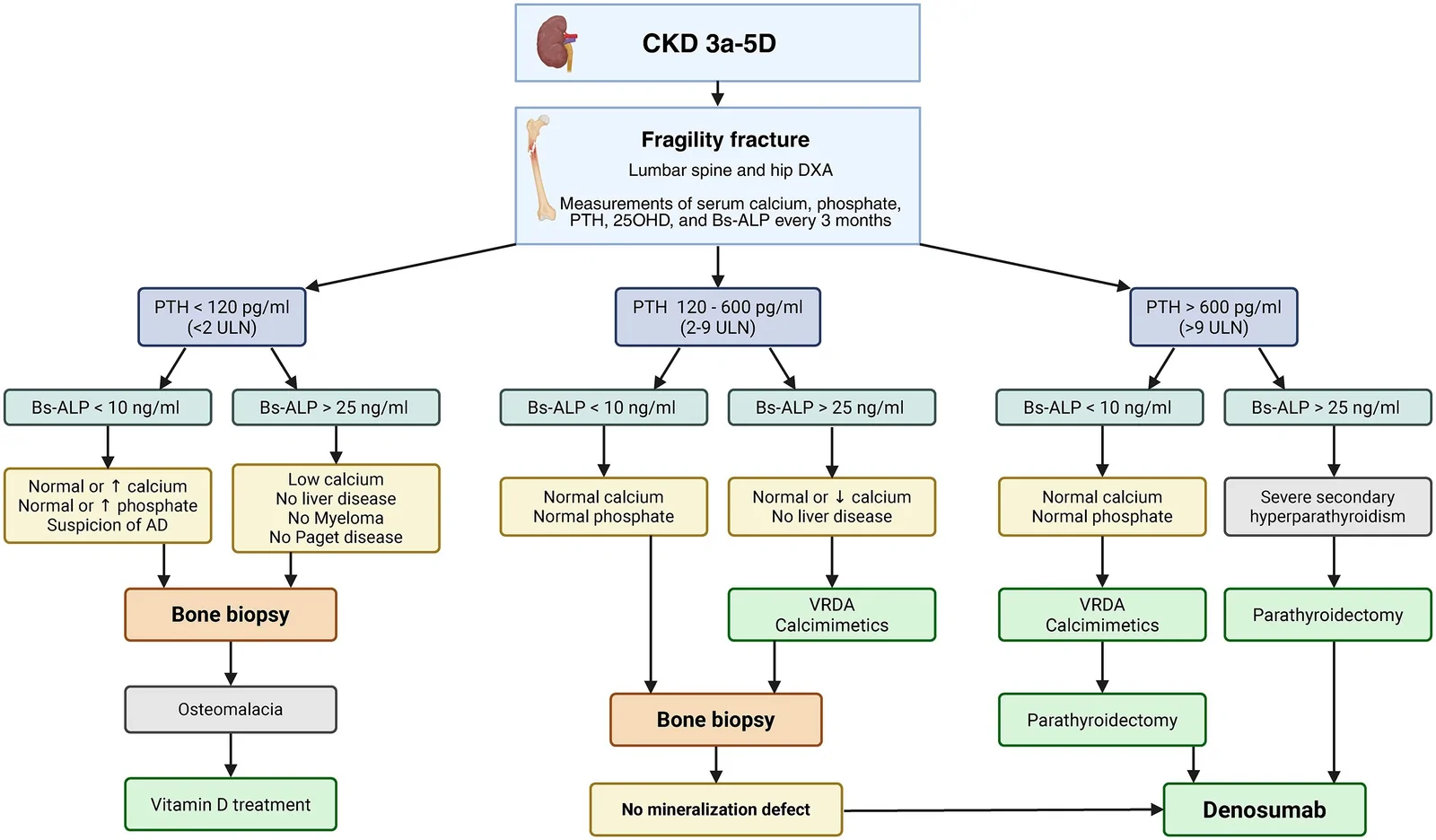
Role of bone biopsy and denosumab use in patients with advanced CKD and fragility fractures. Adapted from Pimentel *et al*. [10]. Created in BioRender. Batteux, B. (2026) https://BioRender.com/c4ic016. AD, adynamic bone disease.

Regarding bone biomarkers, we observed an increase in serum PTH during the first 4–6 weeks after denosumab initiation, likely due to a compensatory parathyroid response to denosumab-induced hypocalcemia from reduced osteoclast activity and bone resorption. However, after 1.8 years, serum PTH decreased in the denosumab group. The absence of a sustained increase in PTH may be due to appropriate vitamin D supplementation before denosumab. Proper calcium/vitamin D supplementation and control of SHPT according to KDIGO targets before starting denosumab are crucial. The observed decrease in P1NP and Bs-ALP in the denosumab group further supports reduced bone remodeling.

The main strength of our study is that the patients were managed in real-life conditions according to European Calcified Tissue Society and the European Renal Association of Nephrology Dialysis and Transplantation guidelines [10]. Our study includes comprehensive bone data with initial histomorphometric, repeated DXA, HRpQCT, and biological data. None of our denosumab-treated patients had biomarkers indicating an overt osteomalacia before treatment, as shown by bone biopsies in seven of nine treated patients. Bone histomorphometric analysis remains the gold standard for classifying CKD-associated osteoporosis, using turnover rate, mineralization, and bone volume as per KDIGO [22]. The promising time-lapse HRpQCT imaging technique is being developed as a substitute for quantitative histomorphometry, but currently, no imaging technique can precisely replace histomorphometry, especially for dynamic labeling [38]. Another strength is using HRpQCT as a noninvasive, low dose, high-resolution tool that discriminates between bone compartments and avoids some DXA artifacts. The use of MMRMs allowed us to account for variation trajectories and different measurement times for HRpQCT parameters.

However, our study has several limitations. The relatively small sample size may have constrained our ability to detect statistically significant positive effects of denosumab on cortical bone or to thoroughly assess its impacts on incident fractures. For this reason, certain differences in baseline parameters between groups (such as age, Bs-ALP, TRAP-5b, and PTH) did not reach statistical significance but may still be considered clinically meaningful. Additionally, the limited number of patients without mineralization defects (*n* = 7) precludes drawing definitive conclusions regarding the optimal efficacy of denosumab in this subgroup. Denosumab-treated patients were also more likely to have a bone biopsy than controls, as this procedure was required prior to therapy initiation. Although HRpQCT offers high-resolution insights into bone microarchitecture, its limited availability in routine clinical setting restricts the broader applicability of our findings. The trabecular bone score (TBS) may represent an alternative tool for monitoring bone status in patients undergoing dialysis and treated with denosumab. A recent meta-analysis showed that dialysis patients have significantly lower TBS values compared with controls (mean difference: −0.106; 95% CI: −0.141 to −0.070; *P* < .01), reflecting impaired trabecular microarchitecture. Dialysis patients with prevalent fractures exhibited lower TBS values than those without fractures. However, TBS was not predictive of incident fractures [39]. Furthermore, there was an initial imbalance between groups in terms of fracture history and bone phenotype, reflecting our clinical practice where denosumab use is primary guided by these factors [10, 40]. To minimize this bias, we applied MMRMs adjusted for relevant covariables to account for these differences as rigorously as possible.

Our findings demonstrate that denosumab confers significant improvements in trabecular bone parameters over time in osteoporotic dialysis patients, effectively preventing persistent trabecular bone loss observed in the absence of treatment. Larger, multicenter studies are warranted to clarify its efficacy in reducing fracture incidence and to refine patient selection criteria for maximal therapeutic impact.

## Supplementary Material

sfag182_Supplemental_File

## Data Availability

The replication data and the scripts file supporting the findings of this study are openly available in repository https://entrepot.recherche.data.gouv.fr/ at doi: 10.57745/L14PET.
