# Supplementary material for: Impact of denosumab on bone high-resolution peripheral quantitative computed tomography in hemodialysis patients: a retrospective longitudinal observational study
Source: Clin Kidney J. 2026 Sep 21;19(10):sfag182. doi: 10.1093/ckj/sfag182 (PMC13639486; doi:10.1093/ckj/sfag182)
Supplement: Supplementary file 1 [file sfag182_supplemental_file.docx]

**Supplementary Material: Table of contents**

**Table S1**: Histomorphometric parameters at baseline.

**Table S2**: HRpQCT parameters at baseline.

**Table S3**: DXA parameters at baseline.

**Table S4**: Changes in DXA parameters over time in groups (MMRMs).

**Table S5**: Changes in biochemical parameters over time in groups (MMRMs).

**Figure S1**: Changes in HRpQCT trabecular parameters at distal tibia in groups (MMRM plots).

**Figure S2**: Changes in HRpQCT cortical parameters at distal tibia and radius in groups (MMRM plots).

**Table S1: Histomorphometric parameters at baseline.**

| **Histomorphometric parameters** | **Total population**  **N = 12** | **Denosumab group N = 7** | **No-denosumab group**  **N = 5** | **P-value** |
| --- | --- | --- | --- | --- |
| **Tb.Vol (%)** | 10.5 [10.0 – 13.2] | 12.3 [9.0 – 14.3] | 10.5 [10.5 – 10.6] | 1.000 |
| **OV/BV (%)** | 8.2 [7.1 – 10.6] | 8.3 [6.2 – 10.5] | 8.0 [7.7 – 10.6] | 0.755 |
| **OS/BS (%)** | 53.1 [46.2 – 62.5 | 52.2 [49.7 – 61.2] | 53.9 [42.5 – 81.5] | 0.876 |
| **Ob.S/BS (%)** | 7.54 [5.98 – 9.86] | 9.48 [4.46 – 10.74] | 7.24 [6.49 – 7.84] | 0.755 |
| **Oc.S/BS (%)** | 1.80 [0.59 – 3.44] | 2.95 [0.94 – 3.50] | 1.27 [0.71 – 1.96] | 0.639 |
| **N.Oc/T.A (/mm²)** | 1.80 [0.60 – 2.88] | 2.87 [1.04 – 3.90] | 1.58 [0.70 – 1.75] | 0.343 |
| **dLS/BS (%)** | 1.26 [0.00 – 8.52] | 2.53 [0.315 – 10.88] | 0.00 [0.00 – 2.91] | 0.503 |
| **Total LS/BS (%)** | 9.70 [2.97 – 19.16] | 9.24 [5.23 – 22.57] | 9.70 [1.12 – 13.79] | 0.714 |
| **dLS (µm)** | 0.00 [0.00 – 4.17] | 2.22 [0.00 – 4.93] | 0.00 [0.00 – 1.47] | 0.426 |
| **MAR (µm/day)** | 0.00 [0.00 – 0.42] | 0.22 [0.00 – 0.49] | 0.00 [0.00 – 0.34] | 0.426 |
| **BFR/BS (µm²/µm3/day)** | 1.86 [0.00 – 4.29] | 2.94 [0.63 – 5.58] | 0.00 [0.00 – 2.46] | 0.312 |

**The results are reported as median [interquartile range].**

***BFR/BS, bone formation rate; dLS, distance between the double labels; ; dLS/BS, double labelled surface; MAR, mineral apposition rate; N.Oc/T.A, osteoclast number; Ob.S/BS, osteoblast surface volume; Oc.S/BS, osteoclast surface volume; OS/BS, osteoid surface volume; OV/BV, osteoid volume; Tb. Vol, trabecular volume; Total LS/BS, total labelled surface.***

**Table S2: HRpQCT parameters at baseline.**

| HRPQCT parameters | Total population  N = 19 | Denosumab group  N = 9 | No-denosumab group  N = 10 | P-value |
| --- | --- | --- | --- | --- |
| Tibia |  |  |  |  |
| BV/TV (%) | 9.6 [7.1 – 11.3] | 9.8 [6.7 – 12.1] | 9.0 [7.7 – 11.0] | 0.842 |
| Dtrab (mg/cm^3^) | 115.1 [85.1 –136.0] | 110.9 [79.9 – 145.3] | 108.2 [92.6 – 131.8] | 0.842 |
| Dcomp (mg/cm^3^) | 667.6 [611.9 – 730.9] | 650.7 [620.6 – 727.9] | 685.3 [597.6 – 732.1] | 0.720 |
| CtTh (mm) | 0.65 [0.42 – 0.78] | 0.49 [0.41 – 0.66] | 0.68 [0.45 – 0.82] | 0.437 |
| CtPm (mm) | 111.7 [103.5 [118.9] | 104.0 [98.7 – 114.5] | 115.7 [105.8 – 119.8] | 0.182 |
| TbN (1/mm) | 1.68 [1.41 – 1.95] | 1.83 [1.68 – 1.99] | 1.57 [1.26 – 1.71] | 0.369 |
| TbTh (mm) | 0.06 [0.05 – 0.06] | 0.05 [0.05 – 0.06] | 0.06 [0.05 – 0.07] | 0.141 |
| TbSp (mm) | 0.53 [0.45 – 0.66] | 0.51 [0.43 – 0.54] | 0.58 [0.52 – 0.73 | 0.278 |
| Tb1/N.SD (tmn) | 0.25 [0.22 – 0.49] | 0.24 [0.20 – 0.27] | 0.27 [0.24 – 0.59] | 0.540 |
| Radius |  |  |  |  |
| BV/TV (%) | 9.1 [5.6 – 11.5] | 8.6 [5.6 – 9.5] | 10.3 [5.8 – 13.8] | 0.307 |
| Dtrab (mg/cm^3^) | 109.4 [67.0 – 138.0] | 102.66 [66.6 – 114.5] | 123.4 [69.0 – 165.6] | 0.278 |
| Dcomp (mg/cm^3^) | 685.2 [620.3 – 723.1] | 702.7 [624.0 – 738.0] | 673.2 [624.1 – 711.8] | 0.842 |
| CtTh (mm) | 0.47 [0.35 – 0.54] | 0.48 [0.31 – 0.53] | 0.45 [0.36 – 0.62] | 0.802 |
| CtPm (mm) | 75.6 [66.7 – 83.7] | 75.0 [66.1 – 77.2] | 78.9 [71.7 – 85.0] | 0.236 |
| TbN (1/mm) | 1.57 [1.31 – 1.82] | 1.67 [1.22 – 1.77] | 1.57 [1.34 – 1.88] | 0.604 |
| TbTh (mm) | 0.05 [0.05 – 0.06] | 0.05 [0.05 – 0.06] | 0.05 [0.04 – 0.07] | 0.935 |
| TbSp (mm) | 0.53 [0.49 – 0.73] | 0.54 [0.50 – 0.77] | 0.51 [0.47 – 0.71] | 0.327 |
| Tb.1/N.SD (tmn) | 0.35 \|0.25 – 0.56] | 0.24 [0.26 – 0.66] | 0.36 [0.22 – 0.50] | 0.447 |

**The results are reported as median [interquartile range].**

***BV/TV, ratio of bone volume to total volume; CtPm, cortical perimeter, CtTh, cortical thickness; Dcomp, cortical volumetric density; Dtrab, trabecular volumetric density; HRpQCT, high-resolution peripheral quantitative computed tomography, Tb.1/N.SD, trabecular distribution; TbN, trabecular number; TbSp, trabecular separation, TbTh, trabecular thickness.***

**Table S3: DXA parameters at baseline.**

| DXA parameters | Total population  N = 19 | Denosumab group  N = 9 | No-denosumab group  N = 10 | P-value |
| --- | --- | --- | --- | --- |
| Lumbar spine |  |  |  |  |
| BMD (g/cm²) | 0.879 [0.768 – 0.942] | 0.774 [0.736 – 0.888] | 0.911 [0.844 – 1.066] | 0.118 |
| T-score | -2.5 [-3.3 – -2.1] | -2.9 [-3.4 – -2.43] | -2.2 [-2.6 – -1.0] | 0.109 |
| Z-score | -0.9 [-1.7 – -0.4] | -2.1 [-2.1 – -0.8] | -0.5 [-1.2 – -0.2] | 0.110 |
| Femoral neck |  |  |  |  |
| BMD (g/cm²) | 0.663 [0.564 – 0.755] | 0.645 [0.587 – 0.689] | 0.697 [0.569 – 0.846] | 0.607 |
| T-score | -2.5 [-3.4 – 2.2] | -2.7 [-3.2 – -2.4] | -2.4 [-3.4 – -1.3] | 0.665 |
| Z-score | -1.1 [-1.6 – -0.4] | -1.4 [-1.7 – -0.8] | -0.8 [-1.4 – -0.1] | 0.243 |
| Total Hip |  |  |  |  |
| BMD (g/cm²) | 0.690 [0.612 – 0.782] | 0.682 [0.614 – 0.695] | 0.764 [0.617 – 0.967] | 0.237 |
| T-score | -2.6 [-3.2 – -2.0] | -2.7 [-3.2 – -2.6] | -2.0 [-3.2 – -0.4] | 0.230 |
| Z-score | -0.1 [-1.8 – -0.6] | -1.7 [-2.3 – -0.9] | -0.9 [-1.2 – +0.3] | 0.203 |
| One-third radius |  |  |  |  |
| BMD (g/cm²) | 0.544 [0.524 – 0.657] | 0.577 [0.508 – 0.657] | 0.545 [0.535 – 0.598] | 0.686 |
| T-score | -4.0 [-4.4 – -2.8] | -3.7 [-4.6 – -2.8] | -4.0 [-4.1 – -3.6] | 0.686 |
| Z-score | -2.8 [-3.6 – -2.0] | -3.4 [-3.8 – -2.7] | -2.3 [-2.8 – -1.7] | 0.343 |
| Ultradistal radius |  |  |  |  |
| BMD (g/cm²) | 0.289 [0.252 – 0.414] | 0.289 [0.257 – 0.355] | 0.325 [0.239 – 0.419) | 0.886 |
| T-score | -4.3 [-4.7 – -2.1] | -4.3 [-4.6 – -3.3] | -3.6 [-5.0 – -2.0] | 1.000 |
| Z-score | -2.7 [-3.4 – -1.6] | -2.5 [-3.6 – -3.0] | -2.1 [-2.6 – -1.4] | 0.200 |

**The results are reported as median [interquartile range].**

***BMD, bone mineral density; DXA, dual-energy x-ray absorptiometry.***

**Table S4: Changes in DXA parameters over time in groups (MMRMs).**

|  |  | **Intra-group analyses** | | | | |  | **Inter-group analyses**  **Denosumab group vs. No-denosumab group** | | | | |
| --- | --- | --- | --- | --- | --- | --- | --- | --- | --- | --- | --- | --- |
|  |  | **Denosumab group**  **N=9** | |  | **No-denosumab group**  **N=10** | |  | **Non-adjusted MMRM** | |  | **IPTW-MMRM** | |
| **DXA parameters** |  | **Estimate (95%CI)*** | **P-value** |  | **Estimate (95%CI)*** | **P-value** |  | **Estimate (95%CI)**** | **P-value** |  | **Estimate (95%CI)**** | **P-value** |
| **Lumbar spine** |  | **N’=7** |  |  | **N’=8** |  |  |  |  |  |  |  |
| BMD |  | 1.02 (0.32 – 1.72) | 0.010 |  | -0.89 (-3.80 – 2.03) | 0.461 |  | 1.91 (-0.51 – 4.33) | 0.141 |  | 2.59 (2.01 – 3.05) | 0.023 |
| T-score |  | 0.06 (0.01 – 0.11) | 0.050 |  | -0.04 (-0.22 – 0.15) | 0.661 |  | 0.10 (-0.08 – 0.27) | 0.248 |  | 0.14 (-0.05 – 0.20) | 0.080 |
| Z-score |  | 0.09 (0.04 – 0.15) | 0.003 |  | -0.01 (-0.24 – 0.22) | 0.929 |  | 0.10 (-0.09 – 0.30) | 0.304 |  | 0.09 (0.00 – 0.21) | 0.327 |
| **Total Hip** |  | **N’=8** |  |  | **N’=7** |  |  |  |  |  |  |  |
| BMD |  | 1.66 (0.81 – 2.52) | 0.001 |  | -3.16 (-5.12 – -1.20) | 0.009 |  | 4.83 (2.92 – 6.73) | <0.001 |  | 4.17 (4.14 – 4.38) | <0.001 |
| T-score |  | 0.06 (0.01 – 0.11) | 0.030 |  | -0.09 (-0.20 – 0.03) | 0.143 |  | 0.14 (0.03 – 0.25) | 0.017 |  | 0.08 (-0.03 – 0.10) | 0.106 |
| Z-score |  | 0.13 (0.08 – 0.17) | <0.001 |  | -0.15 (-0.31 – 0.01) | 0.096 |  | 0.28 (0.14 – 0.41) | <0.001 |  | 0.26 (0.25 – 0.27) | <0.001 |
| **Femoral neck** |  | **N’=8** |  |  | **N’=7** |  |  |  |  |  |  |  |
| BMD |  | 1.53 (0.35 – 2.77) | 0.022 |  | -1.66 (-4.71 – 1.39) | 0.308 |  | 3.25 (0.38 – 6.09) | 0.037 |  | 3.09 (2.48 – 3.69) | 0.034 |
| T-score |  | 0.07 (0.02 – 0.12) | 0.022 |  | -0.08 (-0.24 – 0.08) | 0.369 |  | 0.15 (0.00 – 0.29) | 0.060 |  | 0.14 (0.09 – 0.17) | 0.049 |
| Z-score |  | 0.12 (0.07 – 0.18) | <0.001 |  | -0.05 (0.26 – 0.16) | 0.660 |  | 0.17 (-0.01 – 0.35) | 0.067 |  | 0.16 (-0.05 – 0.23) | 0.065 |
| **One-third radius** |  | **N’=5** |  |  | **N’=2** |  |  |  |  |  |  |  |
| BMD |  | 1.97 (-1.03 – 4.86) | 0.209 |  | -0.62 (-0.81 – -0.43) | <0.001 |  | NA |  |  | NA |  |
| T-score |  | 0.12 (-0.04 – 0.27) | 0.162 |  | -0.04 (-0.04 – -0.03) | <0.001 |  | NA |  |  | NA |  |
| Z-score |  | 0.20 (0.04 – 0.34) | 0.022 |  | -0.02 (-0.01 – -0.04) | <0.001 |  | NA |  |  | NA |  |
| **Ultradistal radius** |  | **N’=5** |  |  | **N’=2** |  |  |  |  |  |  |  |
| BMD |  | 0.76 (-3.54 – 5.07) | 0.736 |  | -2.18 (-4.56 – 0.21) | 0.115 |  | NA |  |  | NA |  |
| T-score |  | 0.06 (-0.21 – 0.32) | 0.684 |  | -0.21 (-0.36 – -0.05) | 0.034 |  | NA |  |  | NA |  |
| Z-score |  | 0.14 (-0.13 – 0.41) | 0.329 |  | -0.12 (-0.30 – 0.06) | 0.229 |  | NA |  |  | NA |  |

***BMD, bone mineral density****;* ***DXA, dual-energy x-ray absorptiometry; MMRM, mixed model for repeated measures; N’, number of patients with longitudinal DXA follow-up; NA, not applicable (small number of patients to perform the model).***

***The reported estimates correspond to the time-fixed effect.**

****** The reported estimates correspond to the fixed effect of time x treatment interaction.**

**The median [maximum – minimum] length of DXA follow-up was:**

- **2.9 years [1.1 – 7.2] in the denosumab group *vs* 2.2 years [1.0 – 4.1] in the no-denosumab group at the lumbar spine.**
- **2.7 years [1.1 – 7.2] in the denosumab group *vs* 2.3 years [1.0 – 4.1] in the no-denosumab group at the total hip and femoral neck.**
- **3.0 years [1.1 – 4.0] in the denosumab group *vs* 2.8 years [2.3 – 3.2] in the no-denosumab group at the radius.**

**Table S5: Changes in biochemical parameters over time in groups (MMRMs).**

|  |  | Denosumab group  N=9 | | | |  | No-denosumab group  N=10 | | | |
| --- | --- | --- | --- | --- | --- | --- | --- | --- | --- | --- |
| Biochemical parameters |  | **N’** | **Follow-up (years)**  **med [min – max]** | **Estimate (95%CI)** | **P-value** |  | **N’** | **Follow-up (years)**  **med [min – max]** | **Estimate (95%CI)** | **P-value** |
| Serum ionized calcium |  | 4 | 1.5 [0.3 – 2.5] | 5.42 (0.25 – 10.57) | 0.063 |  | 4 | 2.6 [1.2 – 3.7] | 1.87 (-1.02 – 4.75) | 0.246 |
| Serum calcium |  | 9 | 2.6 [0.3 – 5.6] | 0.73 (-0.88 – 2.31) | 0.377 |  | 8 | 2.9 [1.0 – 7.2] | 3.36 (0.58 – 6.13) | 0.041 |
| Serum phosphate |  | 8 | 2.6 \|0.3 – 5.6] | 7.75 (-3.37 – 19.96) | 0.192 |  | 5 | 2.9 [1.0 – 7.2] | 0.15 ( -11.00 – 10.77) | 0.979 |
| Serum CTX |  | 7 | 2.5 [0.3 – 3.0] | -9.73 (-26.20 – 6.54) | 0.253 |  | 5 | 1.2 [1.0 – 3.7] | 7.48 (-65.23 – 78.63) | 0.842 |
| PTH |  | 6 | 2.2 [0.3 – 2.6] | -19.19 (-32.97 - -4.15) | 0.016 |  | 4 | 2.7 [1.0 – 5.0] | -16.57 (-45.22 – 12.09) | 0.280 |
| tALP |  | 7 | 2.4 [0.3 – 3.0] | -10.32 (-22.36 – 2.23) | 0.114 |  | 2 | 1.6 [1.2 – 2.0] | -9.67 (-20.14 – 0.76) | 0.111 |
| Bs-ALP |  | 7 | 2.5 [0.3 – 5.6] | -11.78 (-20.80 – -2.76) | 0.019 |  | 6 | 1.9 [1.0 – 5.0] | -0.15 (-10.85 – 9.66) | 0.976 |
| TRAP-5b |  | 8 | 2.5 [0.3 – 3.0] | -4.09 (-15.72 – 7.55) | 0.504 |  | 3 | 2.6 [1.2 – 3.7] | -4.09 (-5.29 – 1.62) | 0.504 |
| P1NP |  | 7 | 2.5 [0.3 – 5.6] | -8.92 (-17.09 – 0.24) | 0.043 |  | 4 | 1.9 [1.2 – 3.7] | -8.76 (-16.46 – 1.77) | 0.137 |

***Bs-ALP, bone-specific alkaline phosphatase; CTX, C-terminal telopeptide of type I collagen; N’, number of patients with longitudinal DXA follow-up; P1NP, procollagen 1 intact N-terminal propeptide; PTH, parathyroid hormone; tALP, total alkaline phosphatase; TRAP-5b, tartrate-resistant acid phosphatase 5B.***

**Figure S1: Changes in HRpQCT trabecular parameters at distal tibia in groups (MMRM plots).**

***BV/TV, trabecular bone volume to tissue volume fraction; Dtrab, trabecular volumetric density; IPTW, inverse probability of treatment weighting; MMRM, mixed model with repeated measures; N, number of patients with repeated HRpQCT measurements over time.***

**Figure S2: Changes in HRpQCT cortical parameters at distal tibia and radius in groups (MMRM plots).**

***CtTh, cortical thickness; Dcomp, cortical volumetric bone mineral density; IPTW, inverse probability of treatment weighting; MMRM, mixed model with repeated measures; N, number of patients with repeated HRpQCT measurements over time.***
